# A One Health perspective on mobile genetic elements and associated antibiotic resistance genes in non-human *A. baumannii*

**DOI:** 10.1128/aem.01086-26

**Published:** 2026-09-22

**Authors:** David Vargas-Peralta, Santiago Castillo-Ramírez

**Affiliations:** 1 Facultad de Ciencias, Universidad Nacional Autónoma de México 7180 , Ciudad de México, México; 2 Programa de Genómica Evolutiva, Centro de Ciencias Genómicas, Universidad Nacional Autónoma de México 7180 , Cuernavaca, México; The Pennsylvania State University, University Park, Pennsylvania, USA

**Keywords:** *Acinetobacter baumannii*, mobile genetic elements (MGEs), insertion sequences (IS), transposon, plasmids, sequence type, international clone (IC)

## Abstract

*Acinetobacter baumannii* has become a global public health concern. Although it has traditionally been associated with hospital settings, it has also been detected in companion animals, livestock, and wildlife, as well as in food and environmental sources, particularly aquatic ones. This review provides an overview of some mobile genetic elements (MGEs) (insertion sequences, transposons, and plasmids) within non-human populations of *A. baumannii*. These MGEs have been associated with antimicrobial resistance (AMR) in both human and nonhuman settings. Importantly, the presence of clinically relevant lineages in non-hospital environments suggests circulation between human and non-human populations. The current evidence suggests that non-human populations and their MGEs may act as reservoirs and disseminators of resistance genes. Overall, these findings broaden its ecological spectrum and reinforce the need for a comprehensive One Health approach.

## INTRODUCTION

Antimicrobial resistance (AMR) is one of the major global public health threats. In recent decades, *Acinetobacter baumannii* has become one of the most significant multidrug-resistant (MDR) bacteria in hospital settings (1, 2). Since 2017, the World Health Organization has classified carbapenem-resistant *A. baumannii* as a critical-priority pathogen, a status that remains in the most recent update in 2024, which reflects the urgent need for new antibiotics against this bacterium (3, 4). *A. baumannii* is a frequent cause of nosocomial infections, primarily affecting patients in intensive care units (ICUs), immunocompromised individuals, patients using medical devices (such as catheters), and those with underlying diseases (5).

Several key factors make *A. baumannii* a difficult pathogen to treat. One is its ability to survive for prolonged periods on dry surfaces, increasing the risk of transmission (6). Another important factor is its capacity to acquire resistance genes through mobile genetic elements (MGEs) (7, 8). The MGEs described in *A. baumannii* that are commonly associated with antimicrobial resistance include insertion sequences (IS), transposons, pathogenicity islands, and plasmids (7, 9–11). These elements not only spread within *A. baumannii* populations but can also be transferred between different bacterial species (12), facilitating the dissemination of resistance across various hosts and environments.

Most studies on this bacterium have focused primarily on clinical settings (13, 14); however, in recent years, there has been a notable increase in research on non-human populations of *A. baumannii* (15–17). The distribution of *A. baumannii* is not limited to humans and hospital environments; it has also been identified in companion animals, livestock, and wildlife, as well as in food products, plants, soils, and even aquatic environments (18–24). Given its widespread presence across diverse environmental sources and its ability to be found in non-human hosts, this bacterium could represent a public health concern. This highlights the need for a One Health approach, which recognizes that human health is closely linked to animal and environmental health (17, 25, 26). Importantly, understanding the biology and ecology of non-human populations of *A. baumannii* is essential for more effectively addressing the global health crisis associated with antibiotic resistance (17, 27). It also facilitates the identification of transmission patterns of resistance determinants among environmental, animal, and human populations.

In this context, this review provides an overview of the most studied MGEs, including insertion sequences, transposons, and plasmids present in non-human populations of *A. baumannii*. This information is integrated according to the source of isolation, including companion animals, livestock and food products, wildlife (where information on MGEs is still limited), and aquatic sources.

## ISs AND TRANSPOSONS

IS elements are the simplest mobile genetic elements. They consist of terminal inverted repeats flanking one or two open reading frames, which encode a transposase (28). This enzyme recognizes these repeats, which are essential for the transposition process. Depending on the insertion sequence family, transposition can occur through conservative (cut-and-paste) or replicative mechanisms. Although IS elements exhibit intracellular mobility, they are not self-transmissible and require a self-transmissible vector, such as conjugative plasmids or bacteriophages, for transfer between cells (28, 29). Transposons have a structure similar to that of IS elements but can carry additional genes, such as antibiotic resistance genes (28). Composite transposons (class I) are flanked by copies of IS elements at each end. Complex transposons (class II), in addition to the transposase and terminal inverted repeats, typically include regulatory genes and a resolvase that recognizes the internal *res* site, allowing the resolution of cointegrates. These elements are not self-transmissible (29). Insertion sequences can contribute to antimicrobial resistance by providing strong promoters that increase the expression of resistance genes, particularly *bla* genes that encode β-lactamases. In turn, transposons promote the dissemination of resistance by mobilizing resistance genes between plasmids and chromosomes (28).

### ISs and transposons in *A. baumannii* from companion animals

Several studies have investigated insertion sequences (ISs) and transposons. For example, in Switzerland, seven *A. baumannii* isolates from dogs and cats belonging to international clone II (IC2) were identified. These isolates harbored two insertion sequences: IS*Aba1* upstream of the *bla*_*OXA-66*_ gene and IS*1133*, typically associated with the *aacC2* gene (30), as part of a residual class 1 integron, a genetic element capable of capturing and expressing gene cassettes through a site-specific recombination system (31). Similar findings have been reported in Portugal and France, where isolates from cats and dogs belonging to ST2^Pas^ (IC2) and ST25^Pas^ (IC7) carried chromosomal *bla*_*OXA-23*_ associated with IS*Aba1* within Tn*2006*- and Tn*2008B*-like structures, respectively (32–34). Additionally, in Lebanon, two IS*Aba1*-carrying cat isolates were detected (35) (Table 1).

**TABLE 1:** Mobile genetic elements from different sources

Source	Insertion sequence(s)	Transposon(s)	Plasmid(s)	Country(ies)	Collection date(s)	Reference(s)
Dog	IS*Aba1*, IS*113*, IS*26*, IS*Aba125*, IS*6100*, IS*Vsa3*, IS*CR2*	Tn*2008*, Tn*2006*, Tn*3*, Tn*125*, Tn*6207*, Tn*6172*, Tn*2008B*	Plasmid: 12.6 kb, plasmid: 13.9 kb, plasmid: 11.3 kb, plasmid: 21.1 kb, plasmid: 48.4 kb, pCUVET596.1, pCUVET19-1836.1, pCUVET19-2231.1, pCUVET18-1476.1, pCUVET18-1476.2, pCUVET18-1476.3	France, Germany, Italy, Netherlands, Switzerland, Thailand	2000–2013, 2004–2009, 2004–2010, 2004–2011, 2004–2012, 2004–2015, 2011–2015, 2012, 2013–2018, 2014, 2016–2019, 2018	(30, 34, 36–41)
Cat	IS*Aba1*, IS*113*, IS*26*, IS*Aba12*, IS*15*, IS*17*, IS*CR2*	Tn*2006*, Tn*2008*, Tn*3*, Tn*6172*, Tn*2008B*	Plasmid: 11.3 kb, plasmid: 12.6 kb, plasmid: 13.9 kb, plasmid: 21.1 kb, plasmid: 55.8 kb, plasmid: 41.2 kb, plasmid: 71.3 kb, pOXA-23-IHIT7853, pCUVET18-1296.1, pCUVET18-833.1, pCUVET18-833.2, pCUVET19-2269.1, pCUVET16-467.1, pCUVET16-467.2, pCUVET16-467.3, pCUVET16-467.4, pCUVET16-467.5, pCUVET18-1051.1, pCUVET18-1416.1, pCUVET19-2090.1, pCUVET19-483.1, pCUVET19-483.2, pCUVET19-483.3, pCUVET18-1335.1, pCUVET18-1335.2, pCUVET18-1335.3, pCUVET19-2158.1, pCUVET19-2158.2, pCUVET19-2158.3, pCUVET18-1740.1, pCUVET18-1740.2, pCUVET18-1740.3, pCUVET18-414.1, pCUVET18-414.2, pCUVET18-414.3, pCUVET18-414.4, pCUVET18-414.5, pCUVET18-414.6, pCUVET18-414.7	France, Germany, Italy, Lebanon, Portugal, Switzerland, Thailand	2000–2013, 2004–2013, 2004–2014, 2009, 2011–2015, 2012–2013, 2013–2018, 2014, 2015, 2016–2019, 2022	(30, 32–37, 39, 41, 42)
Sheep	IS*Aba1*, IS*Aba19*, IS*26*, IS*Pa14*, IS*Aba125*, IS*Aba12*	Tn*6171*, Tn*1213*, Tn*2008*, Tn*aphA6*, Tn*6022*-delta-1, Tn*6172*	pAbPK1a, pAbPK1b	Pakistan	2011–2012, 2012	(43, 44)
Horse	IS*Aba1*	Tn*2008*		Belgium	2012	(45)
Dairy cattle		Tn*2008*		France	2010	(46)
Bird		Tn*801*, Tn*7*, Tn*4*		Brazil	2012	(44)
Duck		Tn*aphA6*, Tn*2*, Tn*801*, Tn*402*	p29FS20−1, p29FS20−2, p31FS3-2-1	China	2017, 2019	(44, 47)
Axillary seabream/ Spanish seabream		Tn*2006*		Algeria	2012	(48)
Wastewater	IS*Aba1*, IS*Aba125*, IS*1396*, IS*Aba124*, IS*Aba24*	Tn*2006*, Tn*2009*, Tn*2006*-like, Tn*125*-like, Tn*7382*-like, Tn*2008*, Tn*As3*	pMAL1, pA105-like	Brazil, China, India, Nigeria, Poland, Romania	2010, 2018, 2019, 2019–2022, 2022, 2023	(49–54)
Seine river	IS*Aba1*			France		(55)
Gray parrot			pIHIT32296	Luxembourg	2016	(56)
Pig	IS*Aba125*	Tn*3*	pNDM-AB, pAB18PR065	China	2011–2012, 2018	(57, 58)
Frozen turkey liver	IS*Aba19*	Tn*3*-like	pEC_gr13, pEC_gr6, pEC_mcr4.3	Brazil	2017	(59)
Powdered milk			Col440II, Col440I, ColRNAI	Germany		(60)
Guayule retting process	IS*Aha3*, IS*Aba26*, IS*Aba13*, IS*Aba44*, IS*Aha2*, IS*1006*, IS*Aba45*, IS*17*, IS*1008*, IS*Aba22*, IS*Aba4*, IS*ABA31*, IS*Aba2*, IS*Aba46*, IS*1007*, IS*Acsp3*		pAbNCIMB8209_134	Australia	2019	(61)
Veterinary clinic			Plasmid: 12.6 kb, plasmid_rep7a	Netherlands, Germany	2013–2018, 2014	(39, 40)

A study analyzing companion animals identified 58 *A. baumannii* isolates carrying IS*Aba1* upstream of *bla_OXA-51_*. The *bla*_*OXA-23*_ gene was also detected in three isolates: one from a cat (ST1^Pas^/ST231^Oxf^, IC1) and two from dogs (ST10^Pas^/ST585^Oxf^, IC8), associated with IS*Aba1* within the transposon Tn*2008* (36). In Italy, five *A. baumannii* isolates with an extensively drug-resistant (XDR) phenotype were identified in hospitalized dogs and cats. These isolates carried three resistance genes: *bla_OXA-23_* within Tn*2006*, *bla_ADC-73_* associated with *ISAba1*, and *bla_TEM-1D_* in a Tn*3*-derived structure flanked by IS*26* (IS*26*–*bla_TEM-1D_*–Tn*3*–IS*26*). Notably, these strains remained susceptible to colistin (37).

Remarkably, in a dog isolate belonging to ST25^Pas^ (IC7), *bla_NDM-1_* (a metallo-β-lactamase) was identified within Tn*125* flanked by IS*Aba125*. Since this transposon is one of the main vehicles for *bla_NDM-1_* in Acinetobacter and Enterobacterales of human origin, its presence suggests a possible incidental acquisition from humans to companion animals (38).

Of note, the presence of similar clonal lineages (IC1, IC2, IC7, and IC8) in both humans and companion animals suggests transmission between these sources (17). Under this scenario, the potential spreading of *A. baumannii* between pets and humans raises the possibility that these animals may act as reservoirs and potential disseminators of insertion sequences and transposons associated with antimicrobial resistance.

### ISs and transposons in *A. baumannii* from livestock and food products

In sheep, an *A. baumannii* strain belonging to ST2^Pas^ (IC2) was identified as the causative agent of a pneumonia outbreak and was found to harbor a wide variety of MGEs, including plasmids, genomic islands, transposons, and insertion sequences (43). Multiple ISs were identified, including IS*Aba1*, IS*Aba19*, IS*26*, IS*Aba125*, IS*Aba12*, and IS*Pa14*, several of which were associated with transposons and antibiotic resistance genes. For example, IS*Aba125* flanks Tn*aphA6*, which carries *aph(3′)-VIa*; IS*Pa14* mediates the integration of *bla_PER-1_* into Tn*1213*; and Tn*2008* carries *bla_OXA-23_*, which is located in both the chromosome and the plasmid pAbPK1b. In addition, Tn*6171* is associated with a gene cluster involved in siderophore biosynthesis and transport (43). In contrast, a study analyzing 16 susceptible isolates from swine and cattle did not detect resistance islands (RIs), IS*Aba1*, or class 1 integrons (62). There are also studies considering farm animals that have identified Acinetobacter isolates at the genus level associated with MGEs. In a study on horses, two *Acinetobacter* spp. isolates were identified carrying the *bla_OXA-23_* gene associated with IS*Aba1* within the transposon Tn*2008* (45). Similarly, another study conducted in France on dairy cattle identified *Acinetobacter* spp. isolates; in one of them, *bla_OXA-23_* was also associated with IS*Aba1* within Tn*2008*, while in other isolates, the IS*Aba1* sequences were found truncated by IS*Acsp2* (46). Regarding food products, a study conducted in India analyzed isolates obtained from chicken and pork meat and included 51 publicly available genomes from production animals, finding a diversity of transposon types. In total, 11 types of transposons were identified: Tn*1213*, Tn*2008*, Tn*2*, Tn*402*, Tn*6022-Δ1*, Tn*6171*, Tn*6172*, Tn*801*, Tn*aphA6*, Tn*7*, and Tn*4*. The study also identified the *bla*_*OXA-23*_ gene within the transposon *Tn*2008 in a sheep isolate (44) (See Table S1). Table S1 lists the different MGEs found in different non-human strains.

Although evidence in livestock remains limited and is often restricted to the genus level, the recurrent detection of *bla*_*OXA-23*_ associated with IS*Aba1* within Tn*2008* (also identified in human isolates) reinforces the idea of transmission between human and animal sources. Considering this, it is important to note that the inappropriate use of antibiotics in livestock practices may promote the spread of these resistant strains.

### ISs and transposons in *A. baumannii* from wildlife

*A. baumannii* has also been isolated from the axillary seabream (*Pagellus acarne*), a marine fish species from the Mediterranean. The identified isolates belonged to ST2^Pas^ (IC2) and carried *bla*_*OXA-23*_ within the transposon Tn*2006*, located on the chromosome. These findings suggest that such isolates may originate from river contamination driven by human activities, with polluted waters eventually reaching Mediterranean coastal regions (48). In contrast, a study analyzing more than 400 isolates from white storks, soil, earthworms, and other environmental sources identified new sequence types (STs), several of them related to international clones, but without detecting IS*Aba1*/*bla_OXA-51_*-like genetic structures. This suggests that these isolates retain a still-pristine genetic context and provide a point of comparison for studying the evolutionary adaptation of *A. baumannii* to hospital environments and multidrug resistance (63).

### ISs and transposons in *A. baumannii* from aquatic sources

Environmental studies on *A. baumannii* have mainly focused on aquatic environments. In Poland, a study analyzing treated wastewater identified seven *A. baumannii* isolates carrying the *bla_OXA-51_*-like gene associated with IS*Aba1* (49). Similarly, a metagenomic study conducted on wastewater and aquatic environments impacted by urban and industrial activities, such as the Pune River and Kazipally Lake in India, detected *A. baumannii* with the presence of IS*Aba1* (50). A study in China analyzing untreated hospital wastewater identified nine carbapenem-resistant *A. baumannii* isolates. Among these, eight belonged to ST2^Pas^ and carried the transposon *Tn2006* (ΔIS*Aba1*-*bla_OXA-23_*-ATPase-yeeB-yeeA-ΔIS*Aba1*), while one ST131^Pas^ isolate carried *Tn2009* (ΔIS*Aba1*-*bla_OXA-23_*-*ATPase*-*hp*-*parA*-*yeeC*-*hp*-*yeeB*-ΔIS*Aba1*) (51) (Table 1).

In Nigeria, a study of hospital wastewater identified 22 isolates carrying *bla*_*OXA-23*_ within a transposon Tn*2006*-like (IS*Aba1*–methyltransferase–dead helicase–ATPase–*bla_OXA-23_*–IS*Aba1*). Likewise, 24 isolates carrying *bla_NDM-1_* were found to harbor a transposon Tn*125*-like (IS*Aba125*–*PAC*–IS*CR21*–*groEL*–*groES*–*cutA*–*dsbD*–*ISO*–*ble*–*bla*_NDM-1_–IS*Aba125*). In addition, four isolates belonging to ST472^Oxf^ carried a transposon Tn*7382-like* (52); meanwhile, in Brazil, an isolate belonging to ST79^Pas^/ST227^Oxf^ (IC5) was identified carrying *bla*_*OXA-23*_ within the transposon Tn*2008*, with the structure IS*Aba1*–*bla*_*OXA-23*_–*ATPase* (53). In contrast, other studies on wastewater did not detect the presence of IS*Aba1* (64, 65); however, this does not exclude the presence of resistance or virulence genes similar to those observed in clinical strains.

In another study, an *A. baumannii* isolate was obtained from the Seine River in Paris, in which the *bla*_*OXA-23*_ gene was identified on the chromosome and associated with IS*Aba1* (55). All the abovementioned studies suggest that diverse aquatic environmental strains have important beta-lactamase genes associated with ISs and may act as reservoirs and potential sources for the dissemination of resistance genes.

Overall, the available evidence shows that insertion sequences and transposons—particularly IS*Aba1*, Tn*2006*, Tn*2008*, and Tn*125*—are widely distributed in non-human *A. baumannii*, with stronger evidence in companion animals and aquatic environments, although they have also been documented in livestock and their products. In contrast, information from wildlife remains limited. The recurrence of these elements in lineages associated with international clones, especially IC1 and IC2, suggests possible transmission between human and animal reservoirs. From a One Health perspective, these findings highlight the role of animals and aquatic environments as reservoirs and potential routes for bacterial dissemination, which in turn underscores the need to extend surveillance beyond hospital settings to include animal and environmental reservoirs.

## PLASMIDS

Plasmids are extrachromosomal elements capable of autonomous replication that vary in size, genetic complexity, and copy number and can occur in either linear or circular forms. Based on their transfer capacity, they are classified as conjugative, mobilizable, or non-mobilizable; the latter are often large and, in some cases, show evidence of domestication as secondary chromosomes (29, 66). Plasmids promote the dissemination of antimicrobial resistance by mobilizing resistance genes between bacteria, particularly those associated with OXA-type carbapenemases and other multidrug resistance determinants (7, 8), highlighting their importance in the circulation of resistance across human and animal sources.

### Plasmids in *A. baumannii* from companion animals

In a veterinary setting, one study identified 29 isolates, including 28 ST1^Pas^/ST231^Oxf^ (IC1) and 1 ST578^Pas^/ST799^Oxf^, carrying *bla_OXA-58_* on four plasmid types (11.3, 12.6, 13.9, and 21.1 kb). Additionally, 18 ST1^Pas^/ST231^Oxf^ isolates harbored AbaR islands. In the same study, 13 ST10^Pas^/ST447^Oxf^ (IC18), ST25^Pas^/ST229^Oxf^ (IC7), and ST602^Pas^/ST732^Oxf^ isolates carried *bla_OXA-23_*. Among the 13 isolates, four ST25^Pas^/ST229^Oxf^ isolates carried *bla*_*OXA-23*_ on the chromosome within the Tn*2006* transposon, whereas the remaining nine harbored the gene on four plasmid types (41.2, 48.4, 55.8, and 71.3 kb) associated with Tn*2008* (39) (Table 1). It is worth noting that, among the STs mentioned, ST25^Pas^ has been reported in humans, non-human animals, and environmental sources, likely due to a particular configuration of virulence factors (67). In this context, characterizing the MGEs circulating in ST25^Pas^ would be relevant.

In Germany, an ST1^Pas^/ST231^Oxf^ isolate was recovered from a cat with cystitis. The isolate harbored a ~ 54-kb plasmid, designated pOXA-23-IHIT7853, carrying *bla*_*OXA-23*_, *strA*, and *strB*. Additionally, its genome contained five prophage regions and multiple insertion sequences, including IS*Aba1*, IS*Aba12*, IS*15*, IS*17*, and IS*26* (42) (Table S1). In a One Health study, *A. baumannii* isolates were recovered from dogs and environmental surfaces in a veterinary clinic. The six ST2^Pas^ isolates did not harbor any plasmids; however, together they harbored IS*6100*, Tn*6207*, AbaR4, and IS*Vsa3*. In contrast, plasmids were detected in only two environmental isolates: one recovered from the operating table (ST239^Pas^), whose plasmid carried *rep_7a_* and the chloramphenicol resistance gene *cat*_*PC221*_, and another recovered from the preparation room (ST837^Pas^), whose plasmid contained the tetracycline resistance gene *tet*(R) (40).

In one of the most comprehensive studies on plasmids in dogs and cats, conducted in Thailand, isolates of *A. baumannii* and *Acinetobacter pittii* were identified. In *A. baumannii*, isolates from CC2 carried plasmids without resistance genes, whereas in the case of multidrug-resistant CC25 isolates, megaplasmids of ~200 kb were detected. These megaplasmids contained a BREX system (bacteriophage exclusion), Tn*6172*-derived elements involved in the capture and rearrangement of resistance genes, and conjugative capacity. Additionally, several of these megaplasmids lacked identifiable *rep* genes, suggesting novel replication mechanisms (41) (Table S1).

In the same study, an *A. pittii* (ST2842^Pas^/ST1427^Oxf^) isolate from a cat harbored several plasmids carrying resistance genes, including pCUVET18-414.1 (*bla_OXA-23_*), pCUVET18-414.3 (*tet*(39)), pCUVET18-414.5 (*ant*(2″)-Ia), and pCUVET18-414.6 (*aph*(3′)-VI). Conjugation assays demonstrated the transfer of these plasmids to *A. baumannii* NU13R, highlighting the potential of *A. pittii* to act as a reservoir and source of resistance gene dissemination (41). However, this capacity is not limited to *A. pittii*. A study of 837 *Acinetobacter* spp. genomes identified 72 non-baumannii species, including *A. pittii, Acinetobacter nosocomialis, Acinetobacter seifertii*, and *Acinetobacter lactucae* (formerly *Acinetobacter dijkshoorniae*), carrying clinically important resistance genes such as *bla_OXA-23_*, *bla_OXA-24/40_*, *bla_OXA-58_*, and *bla_NDM-1_*. Together, these findings indicate that non-baumannii *Acinetobacter* species serve as reservoirs of resistance genes with the potential to facilitate their dissemination, reinforcing their importance from a One Health perspective (68).

In a study involving a companion African gray parrot (*Psittacus erithacus*), a plasmid from *A. baumannii* (ST294^Pas^) of ~8 kb was identified carrying *bla_OXA-72_*, a Rep protein of the Rep3 family, and a putative type II toxin-antitoxin (TA) system (*VapC2*–*VapB2*), the latter possibly involved in plasmid stability. This finding suggests that non-traditional companion animals may also serve as relevant reservoirs of *A. baumannii* (56) (Table 1).

### Plasmids in *A. baumannii* from livestock and food products

In a study on farm animals, an *A. baumannii* isolate positive for *bla_NDM-1_* was identified from a lung sample of a pig with pneumonia. The gene was located on a ~47-kb plasmid, designated pNDM-AB, organized into three regions: one for conjugation and replication, one encoding a type IV secretion system, and a resistance region harboring *bla_NDM-1_*, *aphA6*, *ble*, and *msr(E)*-*mph(E)*; it also contained a copy of IS*Aba125* (57).

In China, an *A. baumannii* isolate from a pig resistant to colistin and polymyxin B was identified. This isolate carried the *mcr-4.3* gene on a ~25-kb plasmid, designated pAB18PR065. This plasmid was not transferable by conjugation, transformation, or electroporation and contained two copies of type II toxin-antitoxin systems, likely involved in its stabilization and maintenance (58).

A study investigating tigecycline resistance in poultry (ducks and chickens) identified 10 MDR *A. baumannii* isolates belonging to ST355^Oxf^ and ST1980^Oxf^. Whole-genome sequencing of a duck ST355^Oxf^ isolate revealed two plasmids, p29FS20−1 (~6 kb) and p29FS20−2 (~12 kb). The *tet*(X5) gene was located on p29FS20−1 together with genes conferring resistance to β-lactams (*bla_OXA-429_* and *bla*_*ADC-25*_), aminoglycosides (*aph*(3″)-*Ib*, *aph*(6)-*Id*, and *aph*(3′)-*Ia*), phenicols (*floR*), and sulfonamides (*sul2*). Interestingly, conjugation assays demonstrated that the *tet*(X5)-*hp*-*hp*-IS*CR2* cassette was transferred to the rifampin-resistant (RIF-R) recipient strain *Acinetobacter baylyi* ADP1 through the activity of IS*CR2*, suggesting that this cassette can be mobilized among *Acinetobacter* species. In contrast, whole-genome sequencing of another duck isolate belonging to ST1980^Oxf^ identified a plasmid designated p31FS3−2-1 (~6 kb); however, the *tet*(X6) gene was detected on the chromosome rather than on the plasmid (47).

In the Czech Republic, an *A. baumannii* isolate (ST345^Pas^/ST747^Oxf^) obtained from frozen turkey liver imported from Brazil showed colistin resistance mediated by *mcr-4.3*. This gene was located on a non-conjugative, non-transformable plasmid that included an additional ~24-kb segment associated with acyl-CoA, absent in plasmids described in clinical strains. These results highlight the potential role of global food trade in the dissemination of *mcr*-carrying plasmids to humans (59).

In a study on powdered milk, three *A. baumannii* isolates were obtained carrying plasmids with 86%–90% similarity to Col440I-, Col440II-, and ColRNAI-type replicons (60), previously described in food-associated bacteria from the genus *Klebsiella* (69). These findings underscore the remarkable ability of *A. baumannii* to acquire mobile genetic elements from other bacterial species (12) (Table 1).

### Plasmids in *A. baumannii* from wildlife

Although there are some studies on *A. baumannii* in wildlife (20, 63, 70), information on the MGEs circulating in these populations remains limited. In some cases, *Acinetobacter* isolates have been identified only at the genus level, along with certain associated MGEs. For example, a metagenomic study on the decomposition of wild mammal carcasses, using the plateau pika (*Ochotona curzoniae*) as a model, detected changes in the microbial community of soil associated with carcasses (gravesoil), including opportunistic pathogens. Among them, *Acinetobacter* stood out due to its increase, reaching up to 14 times higher abundance than in control soil and showing positive co-occurrence with resistance genes such as *mecA* and *ROB-1*. In addition, the microbial community exhibited a wide repertoire of MGEs, with a predominance of transposases (46.92%), plasmids (35.82%), and IS*91* (8.54%), highlighting the central role of these elements in shaping soil resistance profiles (71). This study suggests that natural environments associated with decomposition may act as reservoirs of antibiotic resistance genes (ARGs) and MGEs, although further research is needed to confirm this pattern across other systems.

### Plasmids in *A. baumannii* from aquatic sources

Numerous studies have shown that aquatic environments act as key reservoirs for the dissemination of plasmids and resistance genes in *A. baumannii* (50, 72–74). In Romania, *A. baumannii* isolates were identified in hospital wastewater, treatment plants, and rivers, including clinically relevant clones such as ST1^Pas^ (IC1) and ST2^Pas^ (IC2). The *bla*_*OXA-72*_ gene was located on a pMAL1-type plasmid, while *bla*_*OXA-23*_ and aminoglycoside resistance genes such as *aph*(3')-*VIa* were found on pA105-type plasmids, particularly common in ST2^Pas^ isolates. Furthermore, conjugation assays demonstrated that the pMAL1-type plasmid could be transferred to the recipient strain *A. baylyi* RIF-R. These findings highlight the role of treatment plants and rivers as dynamic reservoirs where clinical and environmental clones exchange genetic material that may persist even after wastewater treatment processes (54) (Table 1).

A metagenomics study in South Africa analyzed various samples (wastewater, treated effluents, sludge, river water, and sediments), identifying *Acinetobacter* among the most abundant genera, along with other pathogens from the genera *Escherichia*, *Pseudomonas*, and *Staphylococcus* (75). These bacteria shared a core mobilome—defined as the set of mobile genetic elements within a microbiome—including plasmids, transposons, and integrons (76). Although wastewater treatment reduces bacterial loads, effluents and sludge remain important sources of environmental dissemination of resistance (75, 77).

In Australia, a study on *Acinetobacter* isolates from wastewater and a lake analyzed multiple species within the genus. An environmental *A. baumannii* isolate (ST350^Pas^) lacked plasmids but showed a high capacity to acquire clinically relevant plasmids (pRAY and pACICU2). Meanwhile, other species such as *Acinetobacter towneri* and *Acinetobacter johnsonii* carried plasmids with pdif modules containing relevant resistance genes such as *bla*_*OXA-58*_, *tet*(39), and *msr*(E)-*mph*(E), similar to those described in global *A. baumannii* clones (ST1^Pas^ and ST2^Pas^) (73). Together, these results indicate that although *A. baumannii* attracts a lot of attention, other environmental species also actively contribute as reservoirs and mediators in the spread of antimicrobial resistance (73, 78).

Furthermore, the presence of *A. baumannii* is not restricted to clinical or aquatic environments. An example is an isolate (ST1197^Pas^) dating back to 1943, obtained from the guayule retting process. This isolate carried a ~134-kb plasmid with a Rep3 protein, partitioning genes, and toxin-antitoxin systems. Although no resistance genes were detected, the plasmid contained multiple insertion sequences, and the chromosome already harbored *bla*_*OXA-78*_ (OXA-51-type). This isolate provides insights into the natural habitats of this bacterium outside hospital settings and is particularly relevant as it predates the widespread use of antibiotics (61) (Table S1).

The aforementioned studies show that plasmids play a central role in the dissemination of resistance genes in *A. baumannii* across different niches, including clinical, aquatic, animal, and wildlife environments. Although various plasmids and associated mobile genetic elements have been identified, the information remains fragmented, especially outside clinical settings. This underscores the need to expand research into non-clinical contexts, where reservoirs and transfer dynamics may still be underexplored but are crucial for understanding the evolution and spread of antimicrobial resistance.

Available evidence indicates that plasmids play a central role in the dissemination of resistance genes in *A. baumannii* across diverse niches, including clinical, aquatic, animal, and wildlife environments. However, knowledge of their diversity, distribution, and genetic content remains fragmented, particularly outside hospital settings. This underscores the need to expand research into non-clinical environments and other *Acinetobacter* species, whose plasmids may serve as reservoirs and vehicles for gene transfer but remain poorly characterized despite their potential relevance to the evolution and dissemination of antimicrobial resistance.

## FINAL CONSIDERATIONS

In this review, we focused only on ISs, transposons, and plasmids, yet other MGEs have also been described in *A. baumannii*. Among these are integrons, which have been identified not only in human clinical settings (79, 80) but also in animals, including birds and sheep, and environmental sources such as rivers (44, 81). Other MGEs reported in *A. baumannii* include miniature inverted-repeat transposable elements (MITEs) (82), resistance islands (83, 84), and bacteriophages (85, 86). Most of these elements are associated with ARGs in human isolates. However, we do not know to what extent and which MGEs could be associated with virulence traits. Thus, there is a need for efforts that also integrate the study of virulence genes and non-human populations—both animal and environmental—under a One Health approach (15, 17, 87); this would allow a better understanding of how resistance and virulence genes circulate among human, animal, and environmental populations.

In this context, the use of whole-genome sequencing (WGS) has provided several advantages, such as higher typing resolution compared to MLST (88), as well as the characterization of the resistome, virulome, and mobilome of *A. baumannii* (12, 89, 90).

However, there are important biases to consider (91); for instance, much of the available information comes from human isolates and a limited number of countries (26). Another important bias is that, although some studies have applied a One Health approach to compare *A. baumannii* genomes between human and non-human populations (18, 19, 92, 93), these remain limited and generally do not specifically address mobile genetic elements, with a few exceptions such as studies focused on prophages (94, 95).

In the examples here discussed, most of the data are concentrated on companion animals and aquatic sources, leaving aside other non-human populations such as livestock, food, and wildlife. It is important to include these populations as they may harbor variants of antibiotic resistance genes (63) and novel virulence genes (96) that could be associated with insertion sequences, transposons, and plasmids, whether newly identified or already described. Importantly, the use of approaches such as metagenomics would allow the study of *A. baumannii* across different niches (23), facilitating not only the characterization of the resistome, virulome, and mobilome but also the analysis of associated microbial communities (75). This would make it possible to investigate the dissemination of genes at both intra- and interspecies levels.

Several strategies have been proposed to contain antibiotic resistance, which can be broadly classified into two complementary approaches (97). The first focuses on preventing the emergence and spread of resistance through stronger public health policies and the rational use of antimicrobials. These measures include antibiotic stewardship programs, appropriate prescribing practices, the development of new antibiotics, promoting innovation to combat antimicrobial resistance, the implementation of rapid and accurate diagnostic methods, and strengthening epidemiological surveillance systems, particularly in low- and middle-income countries (98). The second approach aims to reduce the environmental burden of resistant bacteria, antibiotic resistance genes (ARGs), and mobile genetic elements (MGEs), particularly in wastewater, to limit their dissemination (97, 99). To achieve this, a variety of treatment technologies have been developed, including enzymatic degradation of extracellular MGEs (99), disinfection and advanced oxidation processes (97), membrane-based separation systems (100), nanomaterials (101–104), and sludge treatment technologies (105), all designed to reduce the persistence and environmental spread of these resistance determinants. Together, both approaches are complementary and essential to containing the emergence and spread of antimicrobial resistance.

In conclusion, the current evidence indicates that *A. baumannii* can no longer be considered exclusively a hospital pathogen but rather a bacterium with a complex ecological dynamic involving multiple hosts and environments. The human-animal-environment interconnection, the selective pressure associated with human activities, and antibiotic resistance gene flow between populations—largely mediated by MGEs such as insertion sequences, transposons, and plasmids—redefine its epidemiological role. In this context, understanding its distribution, adaptation, and dissemination requires moving beyond fragmented approaches and systematically integrating epidemiological, genomic, and ecological data within a truly One Health framework.

## SUPPLEMENTAL MATERIAL

Table S1 aem.01086-26-s0001.xlsx Detailed information on the strains described, including their MGEs, STs, ICs, and other relevant features.

ASM does not own the copyrights to Supplemental Material that may be linked to, or accessed through, an article. The authors have granted ASM a non-exclusive, world-wide license to publish the Supplemental Material files. Please contact the corresponding author directly for reuse.
